# The Effect of Food Odor Exposure on Appetite and Nutritional Intake of Older Adults with Dementia

**DOI:** 10.1007/s12603-021-1719-y

**Published:** 2022-01-14

**Authors:** M.H. Verwijs, O. van de Rest, G.-J. van der Putten, L.C.P.G.M. de Groot, Sanne Boesveldt

**Affiliations:** 1 Wageningen University & Research, Human Nutrition and Health, Stippeneng 4, 6708WE, Wageningen, the Netherlands; 2 Department of Nutrition, Dietetics and Lifestyle, HAN University of Applied Sciences, Kapittelweg 33, 6525 EN, Nijmegen, The Netherlands; 3 Orpea, Dagelijks Leven, Medical Department, Kanaal Zuid 145, 7327 AA, Apeldoorn, The Netherlands

**Keywords:** Olfaction, cognition, nutrition, dementia

## Abstract

**Objectives:**

Dementia can lead to decreased appetite and nutritional intake. Food odor exposure has been shown to increase appetite and nutritional intake in young healthy adults. This study investigates the effect of food odor exposure on appetite, nutritional intake and body weight of Dutch nursing home residents with dementia.

**Design:**

This was a one-armed, non-randomized, non-blinded intervention study consisting of a four-week control period followed by a twelve-week intervention period.

**Setting:**

Four nursing homes in the Netherlands.

**Participants:**

Forty-five nursing home residents with dementia.

**Intervention:**

During the intervention period, odors were dispersed prior to the main meals.

**Measurements:**

General and specific appetite for sweet and savory foods was measured weekly. Nutritional intake was measured once during the control period and three times during the intervention period through a 3-day food record. Body weight was assessed at the start and end of the control period and at the start, end and halfway the intervention period. Data were analyzed with linear mixed models.

**Results:**

Small changes in general and specific appetite were observed after odor exposure. Overall energy intake did not change during the first four intervention weeks, but increased during the second and third (+118kcal/d, p=0.003 and +122kcal/d, p=0.004). Protein intake and body weight did not significantly change during the study.

**Conclusion:**

In this study, no clinically relevant changes in appetite, nutritional intake and body weight were observed after food odor exposure. Future studies should assess the effect of natural food odors and/or meal-tailored odors on nutritional intake of older adults with dementia.

## Introduction

**O**ver the past centuries, life expectancy of our world population has grown steadily. Although we can enjoy life for a longer period of time, aging is often accompanied by physical and/or mental decline (Andersen et al., 2007, Vestergaard et al., 2015, Harada et al., 2013, Reichman et al., 2010). As a result of this age-related mental decline dementia may develop. Appetite of a patient with dementia often changes over the course of time (5, 6), and due to the decline in mental functioning, patients often forget to eat and drink (7). Consequently, malnutrition is frequently present among older adults with dementia (8).

Sensory cues, such as food odors, can increase appetite and influence food choice (Ramaekers et al., 2014, Zoon et al., 2016, Gaillet-Torrent et al., 2014). An example from everyday life: when you walk past a bakery and smell their freshly baked bread, it triggers your appetite for it. Accordingly, studies among healthy adults showed that exposure to food odors enhances appetite for congruent foods, but not for other foods (Ramaekers et al., 2014, Zoon et al., 2016, Gaillet-Torrent et al., 2014, Ramaekers et al., 2014, Proserpio et al., 2017). For example, exposure to banana odor increased appetite for banana and other sweet foods, and likewise, participants who were exposed to a pear odor were more likely to choose fruity desserts compared to participants in the control condition (11, 12). Although appetite for specific foods is shown to increase after exposure to similar food odors, effects on subsequent dietary intake are inconsistent (10, 11, 14, 15). e.g., participants exposed to a chocolate odor more often chose and consumed sweet, high-energy foods compared to the control condition (13, 15). However, other studies showed no impact of food odor exposure on congruent preferences or intake (14, 16).

Previous studies investigating the effect of odor exposure on appetite and nutritional intake were mainly conducted in healthy younger adults (Ramaekers et al., 2014, Zoon et al., 2016, Gaillet-Torrent et al., 2014, Ramaekers et al., 2014, Proserpio et al., 2017). Thus far, only one study by Sulmont-Rossé et al. (2018) investigated the effect of (repeated) exposure to a meat odor prior to lunches on subsequent food intake in nursing home residents with dementia (17). After the first odor exposure, interest towards the meal enhanced and meat and vegetable intake increased with 25%. However, no effects on interest towards the meal nor effects on food intake were shown after the second odor exposure.

Altogether, results from previous studies appear promising and dispersing odors through vaporizers would be a relatively simple way to increase appetite and nutritional intake in an older population that is at risk of malnutrition. Yet, most studies focused on short-term effects only, while long-term effects are more relevant for real-life application. Therefore, the aim of this study is to investigate the effect of a twelve-week food odor exposure on appetite, nutritional intake and body weight of older adults with dementia.

## Materials & Methods

### Participants

Participants were recruited from psychogeriatric wards of four different nursing homes in the Netherlands, all part of the health care organization Amaris. Exclusion criteria were: aged <65 years, BMI>35 kg/m^2^, residing at a somatic or short-stay ward, in a terminal or vegetative stage, using (par)enteral nutrition or not being able to communicate about their appetite.

### Design

This was a one-armed, non-randomized, non-blinded intervention study, consisting of a four week control period, followed by a twelve week intervention period in which odors were dispersed prior to breakfast, lunch and dinner. The twelve week intervention period consisted of three consecutive blocks of four weeks: I1; I2 and I3. Nutritional intake and appetite were measured during the four week control period and the three intervention blocks. Body weight was measured at the start of the control period (BW1) and at the end of the control/beginning of the intervention period (BW2) and halfway (BW3) and at the end (BW4) of the intervention period.

**Figure 1:**
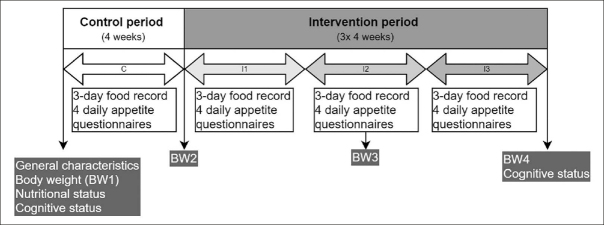
Set-up of the study (C=control period (4 weeks); I1 = First intervention block (4 weeks); I2 = second intervention block (4 weeks); I3 = third intervention block (4 weeks))

In order to reduce any potential seasonal effect on nutritional intake, two nursing homes were included during spring and summer and two during fall and winter. The set-up of the study including all measurements that have been performed is depicted in figure 1.

### Odors

During the twelve week-intervention period odors were dispersed in the communal living rooms or private rooms of the participants, depending on where participants resided during the day. Odors were dispersed three times a day during a period of 30 minutes prior to meal consumption: a bread odor, a vegetable stock odor and a beef stew odor to match breakfast, lunch and dinner respectively. A pilot study among nine older adults was conducted to determine which odors were most pleasant, and best suited for (i.e. be congruent with food consumed during) breakfast, lunch and dinner. All odors were designed and produced by Iscent (Zeewolde, the Netherlands). Odors were dispersed using Iscent 400 vaporizers from the same company. The intensity of the odors was set to be clearly noticeable by healthy adults, therefore likely just noticeable by the participants.

### Measurements

Most measurements were performed on or by the participants themselves. If this was not possible due to memory loss as a result of dementia, e.g. in the case of general characteristics, legal representatives or registered nurses were asked for assistance.

#### General characteristics

Before the start of the study, participant characteristics were recorded. The Dutch version of the Mini Nutritional Assessment Short-Form (MNA-SF) (19) and the Simplified Nutritional Appetite Questionnaire (SNAQ) (20) were administered to assess nutritional status and the risk of losing weight respectively. MNA-SF scores vary from 0 – 14: 0 – 7 indicates undernutrition, 8–11 indicates a risk of undernutrition and scores >11 indicate a normal nutritional status. Outcomes of the SNAQ vary from 0 – 20: outcomes <14 indicates a significant risk of ≥5% body weight loss during the past 6 months. The Severe Impairment Battery (SIB-8) (21) was completed to give insight into participants' cognitive status. The SIB-8 is a non-invasive short questionnaire covering different domains of cognitive functioning. The higher the score on the SIB-8, the better the cognitive status, and vice versa. SIB-8 was measured before the start and at the end of the study.

#### Appetite

General and specific appetite was measured once a week right before breakfast, lunch and dinner during both control and intervention period. In total, appetite was measured four days during the control period and twelve days during the intervention period. Through a 5-point Likert scale (1 = not at all; 2 = not really; 3 = neutral; 4 = a bit; 5 = very) participants were asked to indicate whether they were hungry (general appetite) and to what extent they would like to eat something sweet or something savory (specific appetite: sweet and savory). During the intervention period, the appetite questionnaire was completed after an odor exposure of at least 20 minutes. Data from these appetite ratings were aggregated into one mean per time block, calculated as a mean before breakfast, lunch and dinner, both for general and specific appetite for sweet and savory foods.

#### Nutritional intake

During the study, nutritional intake was monitored through four 3-day food records in total: one 3-day food record during each block of four weeks (control, I1, I2, I3). Research assistants completed the food records on three subsequent weekdays with the help of caregivers. Food records were entered into Compl-Eat, a program that calculates nutritional intake based on the NEVO-database on food composition (RIVM, 2016). Portion sizes of foods and drinks consumed during breakfast, lunch and in-between meals were entered into the food records by means of household measures and standardized portions. Soup, hot meals and desserts were weighed before serving. Possible leftovers were weighed and subtracted from the portion served. Data from the 3-day food records were aggregated into one mean per time block, calculated as a daily total for energy intake (kcal) and protein intake (g).

#### Body weight

Body weight was measured in kilogram (kg) at one decimal accuracy, without shoes or heavy clothing. Wheelchair scales with handrails were used that were available in the nursing homes. In order to calculate body weight, the weight of the wheelchair or walking aid was subtracted from the total weight.

### Data analysis

#### Sample size calculation

Based on the appetite results of a study by Ramaekers et al. (2013) a sample size calculation was performed (9). Using a power of 80% and a two-sided significance level (α) of 0.05 the total required sample size would be 34 research subjects. Anticipating a drop-out rate of ≈20% 40 research subjects in total would be needed to have sufficient research subjects.

#### Analyses

Descriptive statistics were performed and general characteristics are reported as means and standard deviations for continuous data and frequencies and percentages for categorical data. Linear mixed models (intention-to-treat) were performed to test for differences in appetite ratings, energy intake, protein intake, and body weight between control period and the three intervention blocks (I1, I2, I3). For all variables, a two-level structure was used to correct for clustering within the four measurements (Control, I1, I2, I3). Therefore, a random intercept was created at participant level. Time (control/I1/I2/I3) was used as fixed-effect term. Covariates were added to the model when they were significantly correlated with the outcome variable. For energy and protein intake, Age and Height were added to the models as covariates. For body weight, Height was added to the model as a covariate. The outcome of the MNA-SF was added as a covariate in the models of savory appetite before breakfast, before dinner and to the daily total. For sweet and savory appetite before lunch, SIB-score was added to the model as a covariate. Post hoc comparisons (Least Significance Difference) were performed to compare main effects between the different time blocks (control/I1/I2/I3) for appetite ratings, energy intake, protein intake and body weight. Statistical analyses were performed using IBM SPSS Statistics version 25.0 (IBMCorp., Armonk, NY, USA) and a P-value below 0.05 was considered significant.

## Results

### Participants

Forty-five participants living in psychogeriatric wards of four nursing homes were recruited between March 2018 and September 2020. Participants were aged 72 – 98 years (88 ± 6.2), 38 participants were female, and BMI ranged from 17.1 – 34.8 kg/m^2^ (24.4 ± 4.4 kg/m^2^). General characteristics of all participants are depicted in table 1. There were no differences is general characteristics between residents of the four different nursing homes.

**Table 1:** General characteristics of participants in frequencies (percentages); mean values (SDs)

		**Total**
Participants (n)		45
Age		
	Mean (SD)	88.2 (6.2)
	Range	72–98
Gender		
	Male	7 (15.6%)
	Female	38 (84.4%)
BMI		
	Mean (SD)	24.4 (4.4)
	Range	17.1–34.8
Diagnosis		
	Alzheimer's disease	20 (44.4%)
	Vascular dementia	9 (20.0%
	Parkinson's disease related dementia	3 (6.7%)
	Combination of types	6 (13.3%)
	Undiagnosed	7 (15.6)
MNA-SF		
	Mean (SD)	9.4 (2.3)
	Normal nutritional status (12-14p)	7 (15.6%)
	Risk on malnutrition (8-11p)	30 (66.7%)
	Malnourished (0-7p)	8 (17.8%)
SNAQ		
	Mean (SD)	15.4 (2.1)
	No risk	29 (64.4%)
	Risk on 5% weight loss within 6 months (<15p)	16 (35.6%)
SIB score		
	Mean (SD)	13.4 (8.9)
	Range	0–32

Thirty-two participants completed the study: two participants deceased, two participants were not able to finish the study due to physical deterioration and one participant moved to another nursing home during the study. Due to a renovation of the living rooms in one of the nursing homes, data of four participants were collected during fourteen weeks in total (ten weeks of intervention) and data of four other participants were collected during twelve weeks in total (eight weeks of intervention).

### Appetite

#### General appetite

**Figure 2:**
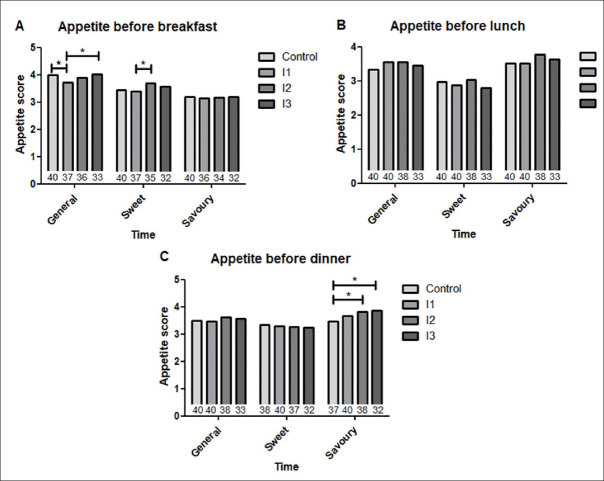
General appetite scores before breakfast, lunch and dinner in control versus intervention blocks (A); Appetite for sweet foods before breakfast, lunch and dinner in control versus intervention blocks (B); Appetite for savory foods before breakfast, lunch and dinner in control versus intervention blocks (C); n is denoted in each bar

Mean general appetite scores ranged from 3.7 – 4.0 before breakfast, indicating a (little) bit hungry, and decreased significantly in I1 compared to the control period (p=0.037). However, general appetite before breakfast returned to baseline level of the control period in I3 (p=0.028). Mean general appetite scores before lunch ranged from 3.3 – 3.6 and from 3.5 – 3.6 before dinner. No significant differences were found for general appetite before lunch and dinner between control and intervention period. Mean appetite scores (±SE) are shown in figure 2A.

#### Appetite for sweet foods

Appetite for sweet foods before breakfast ranged from 3.4 – 3.7 and increased significantly between the first and second intervention block (p=0.042). Appetite for sweet foods before lunch and dinner ranged from 2.8 – 3.0 and 3.2 – 3.3 respectively, but did not change significantly during the intervention period compared to the control period. Mean appetite scores (±SE) are shown in figure 2B.

#### Appetite for savory foods

Mean appetite for savory foods before breakfast and lunch ranged from 3.1 – 3.2 and 3.5 – 3.8 respectively, but did not significantly change during the intervention period compared to the control period. Appetite for savory foods before dinner ranged from 3.5 – 3.9 and increased significantly between the control period and the second (p=0.004) and third intervention block (p=0.001). Mean appetite scores (±SE) are shown in figure 2C.

### Nutritional intake

**Figure 3:**
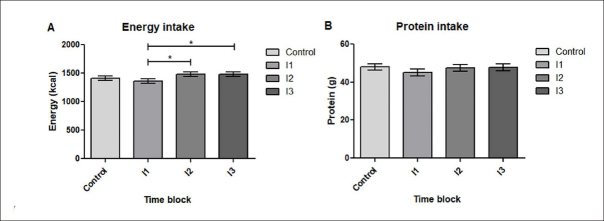
Daily energy intake in kcal during control period and intervention blocks (A); Daily protein intake in grams during control and intervention blocks (B). * indicates significant differences (p<0.05); C n=45, I1 n=43, I2 n=42, I3 n=34

Mean energy intake ranged from 1362 kcal to 1484 kcal per day and mean protein intake ranged from 45.1 and 47.9g/d during the study. As shown in figure 3A, energy intake increased significantly during the second (p=0.003) and third intervention block (p=0.004) compared to the first intervention block. The absolute difference in energy intake was approximately 118 kcal/d between I1 and I2 and 122 kcal/d between I1 and I3. In figure 3B, protein intake throughout the study is shown. There were no significant differences between control period and intervention period, nor between intervention blocks.

### Body weight

**Figure 4:**
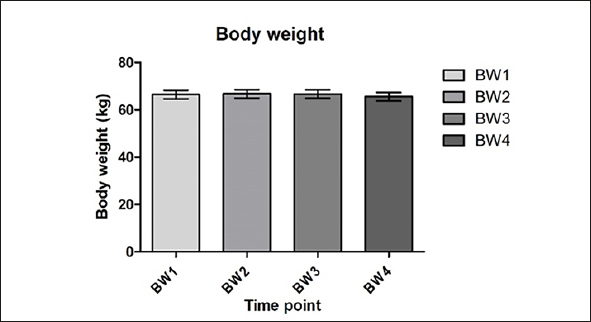
Body weight at the start of the study (C1), after the control period/at the start of the intervention period (C2), halfway the intervention (I1) and at the end of the intervention (I2); C n=41, I1 n=39, I2 n=41, I3 n=35

Mean body weight (kg) ranged from 65.6 – 66.7kg but did not significantly change during the intervention period compared to the control period (figure 4). However, during the last six weeks of the intervention (I2), mean body weight tended to decrease with 1.1kg (p=0.058).

Mean appetite ratings, energy and protein intake and body weight (±SEs) are presented in Supplementary Table S1.

## Discussion

The aim of this study was to investigate the effect of food odor exposure on appetite, nutritional intake and body weight of nursing home residents with dementia. The results of the present study show relatively small effects on general and specific appetite. Energy intake increased significantly during the second and third intervention block compared to the first intervention block, but not compared to the control condition. Protein intake and body weight were not significantly affected during the study. Despite careful consideration of the study design, few clinically relevant outcomes have been achieved during the intervention period. In the following paragraphs we elaborate on potential causes for the absence of clinically relevant outcomes and give recommendations for future research.

Previous studies found that olfactory function is generally impaired at a higher age (24, 25) and in patients with different types of dementia (26). As a result, participants in our study may not have been able to perceive the odors. Consequently, the effect size of the intervention may have increased when the intensity of the odors was adjusted to the olfactory function of individual participants instead of the general intensity maintained in the current study. However, a study by Doorduijn et al. (2020) showed that odor identification is often impaired in patients with dementia (27), while detection thresholds are similar to those of controls. Moreover, the concentration levels of the odors were set rather high, to ensure that all participants were able to detect the odors. Thus, participants in our study may have been able to detect the odors, but they may not have been able to correctly identify them.

Furthermore, a study by Zoon et al. (2016) showed that odors signaling a sweet or savory taste led to an increased appetite for congruent foods (10). As meals were precooked and the menu varied throughout the year it is unlikely that the standard odors used in this study were congruent with all meals consumed subsequent to the odor exposure. In order to optimize the possible effect of food odor exposure on appetite and nutritional intake, future research should match the odors with the meal that is consumed subsequently. Therefore, it would be interesting to study the effect of natural food odors by preparing meals on site.

The absence of a significant effect of odor exposure on our three main outcomes may also be explained by the fact that three standard food odors were dispersed during the twelve-week intervention period, which may have led to olfactory habituation and adaptation (22). Köster et al. (2014) state that humans only respond to novel odors while little attention is paid to known odors (23). Similarly, in a study by Sulmont-Rossé et al. (2018) including older adults with dementia, the second exposure to a food odor did not show any effect on interest towards the meal and the actual meal consumed, while the first exposure to the same odor did positively affect both outcome measures (17). In our study, both appetite and food intake was measured throughout the total period of twelve weeks and not only during the first day of the odor exposure. Therefore, the novelty effect of the odors might have disappeared, showing no significant effect on the main outcomes of our study.

Even though the length of the 12-week intervention period and the fact that the study was performed in a real-life setting were major strengths of this study, the absence of a clinically relevant effect may be explained by the methods to measure these outcomes. Appetite was measured through a 5-point Likert scale. As older adults with dementia may experience difficulties in expressing their needs (28, 29), it is questionable whether the outcomes of the appetite ratings provide reliable outcomes. The method of assessing appetite was different in a study by Sulmont-Rossé et al. (17), i.e. through observations by the experimenters, therefore making it impossible to compare results. Therefore, future research is needed to investigate validity and feasibility of appetite ratings in older adults with dementia. Nutritional intake was measured through 3-day food records where nutritional intake was measured on weekdays by means of observations and by weighing soup, the hot meal and the dessert. This method is shown to be reliable in measuring intake in a population of institutionalized older adults (30) and provides more insights into the effects of food odor exposure on the diet compared to a single meal. However, mean energy intake during the control period (1410 kcal/day) was considerably lower compared to other studies observing nutritional intake in a population of nursing home residents diagnosed with dementia. Previous studies among nursing home residents with mild to severe dementia report an intake of 1650–1789 kcal per day, measured through (weighed) food records (28, 29). This may indicate that energy intake in our population was lower compared to similar populations or that an error measurement has occurred, possibly due to portion estimation as not all meals were weighted. Further, it is recommended to include two weekdays and one weekend-day in a 3-day food record, as food consumption can differ during the weekend. However, in the nursing homes included in this study, there were hardly any differences in the food that was served during weekdays and weekend-days. Therefore, the effect of not including weekend-days should not have caused the large energy difference in our study compared to other studies. Finally, body weight decreased (but not significantly) during the last 6 weeks of the intervention (-1.1kg, p=0.058). This is likely the result of an insufficient energy intake, as shown above, and underlines the urge for sustaining food intake in nursing home residents to prevent malnutrition.

## Conclusion

In conclusion, the results of this study show no clinically relevant effects of food odor exposure on appetite, nutritional intake and body weight of nursing home residents with dementia. Future research should focus on optimizing odors and to validate appetite measurements in this specific population. In this way, further evidence will show whether food odor exposure increases appetite and nutritional intake in older adults with dementia.
