# Supplementary material for: The Effect of Food Odor Exposure on Appetite and Nutritional Intake of Older Adults with Dementia
Source: J Nutr Health Aging. 2022 Jan 14;26(2):112–8. doi: 10.1007/s12603-021-1719-y (PMC12878400; doi:10.1007/s12603-021-1719-y)
Supplement: Supplementary file 1 — Appendix [file mmc1.docx]

## Appendix

| **Supplementary Table S1:** Food intake (energy and protein intake), appetite ratings and body weight during the control and intervention period (I1, I2, I3). | | | | | | | | |
| --- | --- | --- | --- | --- | --- | --- | --- | --- |
|  | Control | | I1 | | I2 | | I3 | |
|  | Mean^a^ | S.E. | Mean | S.E. | Mean | S.E. | Mean | S.E. |
| General appetite |  |  |  |  |  |  |  |  |
| Before breakfast | 4.0 | 0.1 | 3.7*^b^ | 0.1 | 3.9 | 0.1 | 4.0 | 0.1 |
| Before lunch | 3.3 | 0.1 | 3.5 | 0.1 | 3.6 | 0.1 | 3.4 | 0.1 |
| Before dinner | 3.5 | 0.1 | 3.5 | 0.1 | 3.6 | 0.1 | 3.6 | 0.1 |
| Appetite for sweet foods |  |  |  |  |  |  |  |  |
| Before breakfast | 3.4 | 0.1 | 3.4 | 0.1 | 3.7 | 0.1 | 3.6 | 0.2 |
| Before lunch | 3.0 | 0.1 | 2.9 | 0.1 | 3.0 | 0.1 | 2.8 | 0.1 |
| Before dinner | 3.3 | 0.1 | 3.3 | 0.1 | 3.3 | 0.1 | 3.2 | 0.1 |
| Appetite for savory foods |  |  |  |  |  |  |  |  |
| Before breakfast | 3.2 | 0.2 | 3.1 | 0.2 | 3.1 | 0.2 | 3.2 | 0.2 |
| Before lunch | 3.5 | 0.1 | 3.5 | 0.1 | 3.8 | 0.1 | 3.6 | 0.1 |
| Before dinner | 3.5* | 0.1 | 3.7 | 0.1 | 3.8 | 0.1 | 3.9 | 0.1 |
| Energy (kcal) | 1410 | 38.7 | 1362* | 38.9 | 1480 | 39.4 | 1484 | 40.8 |
| Protein (g) | 47.9 | 1.7 | 45.1 | 1.8 | 47.5 | 1.8 | 47.8 | 1.9 |
|  | BW1 | | BW2 | | BW3 | | BW4 | |
|  | Mean | S.E. | Mean | S.E. | Mean | S.E. | Mean | S.E. |
| Body weight (kg) | 66.5 | 1.8 | 66.7 | 1.8 | 66.7 | 1.8 | 65.6 | 1.8 |

Abbreviations: I1, Intervention block 1; I2, Intervention Block 2; I3, Intervention block 3; BW1, before start of control period; BW2, end of control period/start of the intervention, BW3, halfway intervention; BW4, end of intervention period; *, significantly different from I3 (p<0.05); a, Mean values with their standard errors; *b, significantly different from control condition
